# Elucidating the downstream pathways triggered by H_2_S signaling in *Arabidopsis thaliana* under drought stress via transcriptome analysis

**DOI:** 10.1080/15592324.2024.2411911

**Published:** 2024-10-04

**Authors:** Xuefeng Hao, AyyappaKumar Sista Kameshwar, Chonlong Chio, Haiyan Cao, Zhuping Jin, Yanxi Pei, Wensheng Qin

**Affiliations:** a College of Biological Sciences and Technology, Taiyuan Normal University, Jinzhong, China; b Department of Biology, Lakehead University, Thunder Bay, ON, Canada; c Shanxi Key Laboratory for Research and Development of Regional Plants, School of Life Science, Shanxi University, Taiyuan, China

**Keywords:** Arabidopsis thaliana, *lcd/des1-mutants*, drought, abiotic stress, H_2_S signaling mechanism

## Abstract

Hydrogen sulfide (H_2_S) is a crucial signaling molecule in plants. Recent studies have shown that H_2_S plays an equally important role as nitric oxide (NO) and hydrogen peroxide (H_2_O_2_) in plant signaling. Previous studies have demonstrated the involvement of H_2_S in regulating drought and other stressful environmental conditions, but the exact downstream molecular mechanisms activated by the H_2_S signaling molecule remain unclear. In this study, we conducted a comprehensive genome-wide transcriptomic analysis of both wild type (WT) and double mutant (*lcd/des1*). *Arabidopsis thaliana* plants were exposed to 40% polyethylene glycol (PEG) to induce drought stress and 20 µM sodium hydrosulfide (NaHS). The resulting transcriptome data were analyzed for differentially significant genes and their statistical enrichments in the Kyoto Encyclopedia of Genes and Genomes (KEGG) pathways. The results indicated significant upregulation of genes related to photosynthesis, carbon fixation, plant secondary metabolite biosynthesis, inositol and phosphatidylinositol signaling pathways, and stress-responsive pathways in mutant plants under drought stress. Mutant plants with impaired H_2_S signaling mechanisms displayed greater susceptibility to drought stress compared to wild-type plants. In summary, all findings highlight the pivotal role of H_2_S signaling in stimulating other drought-responsive signaling pathways.

## 1. Introduction

Hydrogen sulfide (H_2_S), a foul-smelling chemical compound generated by sulfate-producing microorganisms during anaerobic digestion.^1^ Research on H_2_S is attaching global interest, with a growing number of studies focusing on its role as a signaling molecule in plants and animals.^1,2^ Nitric oxide (NO), hydrogen peroxide (H_2_O_2_), and more recently, H_2_S are acknowledged as important signaling molecules in both plants and mammals.^2^ Within the plant kingdom, H_2_S plays a pivotal role in maintenance various growth and development stages. Specifically, physiological concentrations of H_2_S have demonstrated the ability to enhance seed germination and promote root morphogenesis.^3–5^ H_2_S also facilitates photosynthesis via multiple mechanisms, including promoting chloroplast development, upregulating photosynthetic enzyme expression, and participating in thiol redox modifications.^6^ Collectively, these mechanisms work synergistically to improve the photosynthetic capacity of plants. Moremore, H_2_S effetively mitigates oxidative stress-induced damage through intricate interaction with plant hormones, notably melatonin and ethylene, thereby preserving the structural integrity of chloroplast and elevating the activity of the key enzyme Rubisco, which ultimately enhances photosynthetic efficiency.^7–9^ H_2_S has also been shown to delay aging by modulating mitochondria energy production, inhibiting chlorophyll degradation, and influencing the expression of antioxidant defense and senescence-related genes.^10–13^ Additionally, H_2_S can regulate fruit ripening and senescence processes, whether by counteracting ethylene or modifying antioxidant defense systems.^14,15^ Furthermore, physiological levels of H_2_S bolster plant stress resistance. Much of the research on stress resistance predominantly focus on abiotic stress. H_2_S assists plants in withstanding various abiotic stresses, such as drought, salinity, temperature extremes, hypoxia, and heavy metal toxicity by inducing the expression of stress-related genes, activating endogenous antioxidant systems, and regulating stomatal movements.^16–20^

Drought is the primary abiotic factor affecting annual crop production in agriculture. Recent studies in plant morphology, physiology, and molecular biology underscores the critical role of H_2_S as a signaling molecule in enhancing plant tolerance and resistance to drought-induced stress.^21–25^ Deng et al. (2019) proposed that H_2_S facilitates the uptake of water and nutrients in root systems by modulating root morphogenesis.^4^ Furthermore, H_2_S enhances plant endurance against extended and intensified drought conditions, alleviating damage through osmotic regulation, maintenance of chlorophyll and hormone levels, activation of antioxidant defense mechanisms, and modulating of gene expression associated with drought stress. Duan et al. (2015) reported that H_2_S improves photosynthetic efficiency by regulating the size and density of stomatal pores.^16^ Chen et al. (2011) found that H_2_S promotes chloroplasts biogenesis, expression of photosynthesis-related enzymes, and redox modification of thiol in seedlings.^6^ Researches by Hosy et al. (2003) and Jin et al. (2017) illustrated that H_2_S governs ion flow by managing stomatal closure and mediating changes in osmotic and turgor pressure of guard cells.^26,27^ Additionally, H_2_S plays a principal role in regulating ABC transporters in guard cells to reduce water loss.^28^ During drought conditions, H_2_S alleviates stress and increases osmotic protective agents such as proline and glycine betaine, while also upregulating the expression of genes related to the biosynthesis of polyamines (PAs) and soluble sugars.^23^ Comprehensive studies have shown that H_2_S enhances plant resistance to drought by maintaining the homeostasis of antioxidant enzymes, osmotic regulators, and cysteine.^29^

According to Jin et al., H_2_S effectively delays leaf senescence and maintains favorable conditions for photosynthesis.^11^ Li et al., found that H_2_S mitigates drought-induced damage through both enzymatic and non-enzymatic systems by highly expressing peroxidase, catalase and superoxide dismutase. This leads to the effective clearance of reactive oxygen species, a decrease in malondialdehyde and hydrogen peroxide levels, prevention of membrane lipid peroxidation, and maintenance the structure integrity of membrane proteins and related enzymes.^30^ Thakur et al. (2021) reported that H_2_S can crosstalk with a variety of hormones which participate in the regulation of drought stress in plants.^31^ Recent studies have shown that H_2_S regulates the transcription of abscisic acid (ABA) responsive genes and activates related transcription factors through ABA-dependent stress resistance pathways, leading to increased expression of downstream genes related to drought stress and improved plant tolerance. Shen et al., demonstrated that H_2_S significantly enhances drought tolerance in *Arabidopsis* plants by regulating the expression of miRNA encoding genes *MIR167a, MIR167c, MIR167d, MIR393a* and *MIR396a*, respectively.^22^ Accumulating evidence suggests that H_2_S plays a pivotal role in regulating DNA methylation and protein sulfhydration in plants exposed abiotic stress, ultimately enhancing their stress tolerance.^32–34^ Despite the limited number of studies focusing solely on the epigenetic role of H_2_S in drought stress,^35^ its notable impact on stress resistance hints a potential correlation between H_2_S and epigenetics.

In addition to drought stress, soil salinization is another abiotic stress impacting crop growth and production. Salt stress adversely affects growth and development in various ways similar to drought stress, resulting in osmotic regulation and imbalance, impaired antioxidant system, abnormal stomatal movement and disordered ion dynamic balance, etc. Furthermore, excessive heavy metal pollution in the soil obstructs seed germination, hinders growth and development, disrupts cellular antioxidant and membrane recovery mechanisms, and causes metabolic disorder, etc.^36,37^ As a signaling molecule, H_2_S plays a crucial role in enhancing the plant resistance by triggering several molecular mechanisms that impart resistance from salt, drought and heavy metal pollution. Previous studies have extensively documented the pivotal of H_2_S in bolstering plant resistance to drought, salinity and heavy metal contamination. However, these studies have fallen short in detailing the specific molecular mechanisms triggered by the H_2_S signaling in plants under drought, soil pollution and saline stress. This study aimed to elucidate the molecular mechanisms governed by H_2_S signaling in *Arabidopsis thaliana*. Genome-wide transcriptome sequencing was conducted on *A*. thaliana plants exposed to drought stress, encompassing both wild type (WT) and H_2_S synthesis double mutant (*lcd/des1*), alongside control groups, to uncover the molecular mechanisms influenced by H_2_S signaling under drought stress conditions.

## 2. Materials and methods

### 2.1. Material background and cultivation conditions

This study utilized two varieties of *A*. thaliana: the WT with a Columbia genetic background and a double mutant *lcd/des1* lacking the gene encoding the primary enzyme for H_2_S synthesis. The *lcd/des1* mutant was generated by crossing the T-DNA insertion mutant *lcd* (SALK-082099) with the *des1* mutant (SALK-205358C). *A. thaliana* seeds were sterilized using 75% ethanol for 30 seconds, then exposed to 6% sodium hypochlorite for 10 minutes. After being rinsed three times with sterile water, the seeds were germinated on 1/2 MS medium containing of 1.0% agar, 1.0% sucrose and adjusted to pH 6.0. The cultivation conditions were maintained at a temperature of 23°C, relative humidity of 60%, light intensity of 160 μEmm^−2^s^−1^, and a photoperiod of 16/8 h (light/dark).

### 2.2. Material processing and experimental grouping

The preparation of the polyethylene glycol (PEG) stress medium adhered to the methodology described in the 2006 study by Zhu et al.^38^ Sodium hydrosulfide (NaHS) was utilized as the source of H_2_S, and fumigation procedures were performed in sealed petri dishes to achieve a final concentration of 20 μM. Both PEG stress and NaHS fumigation treatments were concurrently administered for a period of 9 hours. Following the aforementioned cultivation and treatment protocols, 20-day-old *Arabidopsis thaliana* seedlings underwent the subsequent seven treatments (T1–7), respectively: T1: Wild type (WT); T2: *lcd/des1*; T3: WT treated with 20 µM NaHS; T4: WT treated with 40% PEG; T5: *lcd/des1* treated with 40% PEG; T6: WT treated with 40% PEG and 20 µM NaHS; T7: *lcd/des1* treated with 40% PEG and 20 µM NaHS.

### 2.3. Construction and quality control of the library

RNA samples were extracted from seven distinct treatment groups utilizing TRIzol reagent (Life technologies®, USA). After isolation, the purity, concentration, and integrity of the RNA samples were assessed using the Agilent 2100 Bio-analyzer (Agilent Technologies, Inc., USA). The NEBNext® Poly(A) mRNA Magnetic Isolation Module (NEB, E7490, USA) was used to obtain mRNA from high-quality total RNA, followed by fragmentation. The cDNA library was prepared with the NEBNext Ultra RNA Library Prep Kit for Illumina (NEB, E7530, USA) and NEBNext Multiplex Oligos for Illumina (NEB, E7500, USA) according to the manufacturer’s protocol. The collected mRNAs were fragmented into approximately 200 bp RNA inserts, which were used for the first- and second-strand cDNA synthesis. The double stranded cDNA was subjected to end repair by adding an A-tail and adaptor ligation. Subsequently, the segment sizes were selected using AMPure XP beads. The appropriate fragments were isolated using Agencourt AMPure XP beads (Beckman Coulter, Inc., A63881, USA) and further enriched by PCR amplification. After library construction, the concentration and size of the inserted fragments were individually quantified using the Qubit 2.0 and Agilent 2100 platforms. The Q-PCR method was used to precisely determine the effective library concentration, ensuring its quality. Then, high-throughput sequencing was conducted on the HiSeqTM X-ten platform with a read length of 150 bp.

### 2.4. Transcriptome analysis using reference genome-based reads mapping

The raw image data from the Illumina HiSeq X-ten was processed using Illumina’s base calling software to generate raw sequences in FASTQ format. A perl script was used to filter out low-quality reads, which included those with only adaptor, unknown nucleotides > 5%, or Q20 < 20% (indicating a sequencing error rate of < 1%). The filtered clean reads were then aligned to the *A. thaliana* genome (TAIR 9) with Tophat2.^39^ The aligned records produced by the aligners were obtained in BAM/SAM formats and subsequently scrutinized to remove any potential duplicate molecules. Gene expression levels were quantified using FPKM values calculated by the Cufflinks software,^40^ where FPKM represents the number of fragments per kilobase of exon per million mapped fragments.

#### 2.4.1. Identifying differential gene expression

Gene expression analysis was conducted using EBSeq,^41^ with fold change analysis incorporating false discovery rate (FDR) correction through the Benjamini-Hochberg method. This method was utilized to detect and remove false positives and false negatives, reporting only biologically significant differentially expressed genes (DEGs). Genes with a fold change ratio of log2 ≥ 2 and FDR significance score < 0.01 were deemed the most significant DEGs.

#### 2.4.2. Sequence annotation

Genes were compared against various protein database including BLASTX, NCBI non-redundant protein (Nr) database, Swiss-Prot database with a specific cutoff E-value of 10^−5^. Furthermore, genes were searched against the NCBI non-redundant nucleotide sequence (Nt) database using BLASTn with a cutoff E-value of 10^−5^. Genes were then retrieved based on the best BLAST hit (highest score) along with their protein functional annotation. To annotate the genes with gene ontology (GO) terms, the Nr BLAST results were imported into the Blast2GO program.^42^ GO annotations for the genes were obtained by Blast2GO. This analysis mapped all annotated genes to GO terms in the database and subsequently quantified the number of genes associated with each term. Later, a custom perl script was employed to plot GO functional classifications. The obtained annotation was further enriched and refined using TopGo (R package).^43^ The gene sequences were also aligned to the Clusters of Orthologous Group (COG) database to predict and classify functions.^44^ KOBAS^45^ software was employed to test the statistical enrichment of significant DEGs in KEGG pathways.

### 2.5. qRT-pcr analysis and verification

Candidate genes were identified through qRT-PCR. RNA extraction and cDNA synthesis were performed using the PrimeScript™ RT Kit from Takara. qRT-PCR reactions were conducted with SYBR® Premix Ex Taq™ reagents from Takara using the CFX96 Real-Time PCR system from Bio-Rad. The primers for qRT-PCR detection are listed in Table S4. Each qRT-PCR analysis was replicated three times. *Actin* (AT3G46520) was used as the reference gene, and the relative expression level was calculated using the 2^−ΔΔCT^ method.

### 2.6. Data analysis

Data processing and one-way ANOVA were conducted using Excel and SPSS Statistics version 17.0. The data were presented as the mean±standard error, and statistical differences among groups were evaluated using Duncan’s test. Significant differences were indicated by different letters (*p* < 0.05). The graphs were created using SigmaPlot version 10.0 and Adobe Photoshop software.

## 3. Results

### 3.1. Overview of transcriptome data analysis

**Figure 1.**
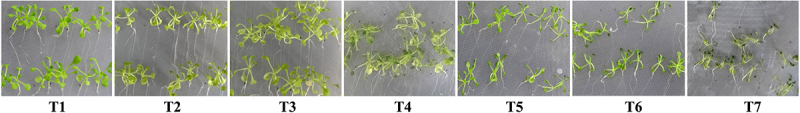
Growth status of 20-day-old *Arabidopsis* seedlings under different treatment conditions. T1: wild type (WT); T2: mutant type (*lcd/des1*); T3: WT + 20 µm NaHS (WT + S); T4: WT + 40% PEG (WT + P); T5: *lcd/des1* + 40% PEG (*lcd/des1* + P); T6: WT + 40% PEG + 20 µm NaHS (WT + P + S); T7: *lcd/des1* + 40% PEG+20 µm NaHS (*lcd/des1* + P + S).

**Figure 2.**
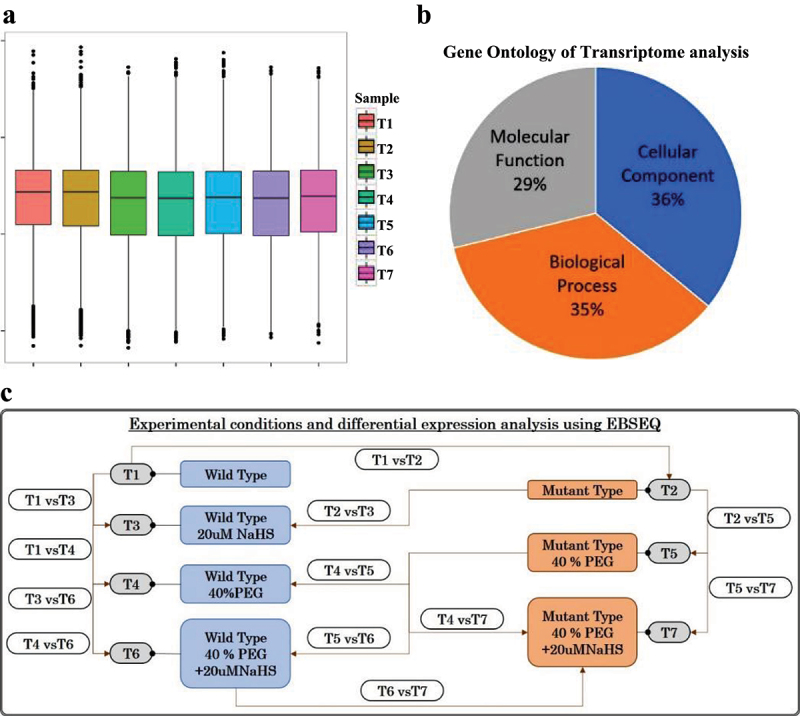
Statistical analysis of gene expression levels and categorization of DEGs using GO annotation under various treatment conditions. (a) Box-whisker plot showing the distribution of expression (FPKM) values of all the sequenced samples (T1 to T7); (b) gene ontology of transcriptome analysis; (c) a graphical depiction of the experimental design employed in this study to facilitate statistical analysis.

To investigate the molecular mechanisms regulated by the H_2_S signaling molecule in both WT and *lcd/des1* mutant *A. thaliana*, which were treated with PEG to artificially mimic drought stress. *A. thaliana* seeds were initially sterilized and cultured on 1/2 MS medium. After 20 days, the seedlings were transferred to PEG stress medium and treated with 20 µM NaHS fumigation to induce H_2_S signaling. The experiment encompassed seven treatments, denoted as T1 through T7, with Figure 1 depicting the respective seedling growth phenotypes observed under each treatment condition. All samples underwent RNA sequencing using Illumina HiSeq X-ten platform. The transcriptomic sequencing yielded an average of 95% reads with >Q30 and an average of 45% GC content (Table S1). Raw reads were then filtered to eliminate low-quality reads and adapter sequences. After alignment, an average of 97% of reads were mapped, with 95% uniquely mapped and 2.6% multiple mapped reads, respectively (Table S2). The annotation of the transcriptome results is based on various annotation databases (Table S3). Gene expression distribution in seven treatment groups was analyzed, as shown in Figure 2(a). Additionally, GO enrichment analysis (Figure 2(b)) was performed. The results demonstrated that 29% of the gene expression was related to molecular function, whereas 36% and 35% were connected to cellular component and biological process, respectively.

### 3.2. Differentially expressed genes in wild type (WT) Arabidopsis thaliana plants under different treatments

**Figure 3.**
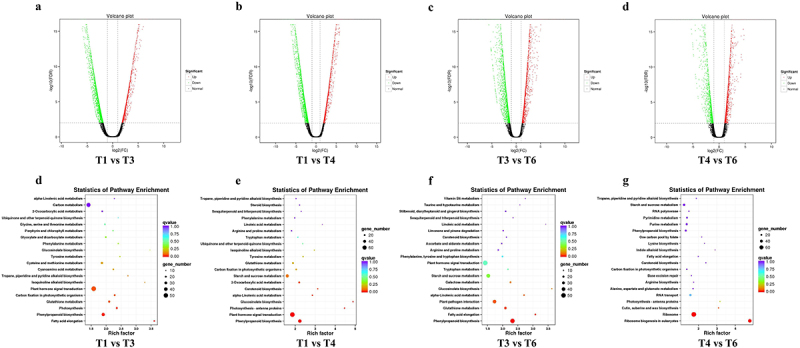
Significantly DEGs and their respective KEGG pathway analyses in response to H_2_S signaling, drought stress, and a combined treatment in wild-type plants. Each point in a volcano plot corresponds to a gene’s differential expression. The horizontal axis depicts the log2 fold change in gene expression between two samples. The vertical axis indicates the negative log10 of the statistical significance associated with the change in gene expression. As the absolute value on the horizontal axis increases, the difference in fold change between the two samples becomes more pronounced. As the value on the vertical axis increases, both the significance of differential expression and the reliability of the selected genes increase. Green points signify genes with down-regulated expression, red points indicate genes with up-regulated expression, while black points represent genes without significant differential expression. The same as below.

To investigate the impact of drought stress-mimicked by PEG and H_2_S signal on wild type *A. thaliana* plants, differential gene expression analysis was conducted using EBSeq,^42^ applying the Benjamini Hochberg false discovery rate (FDR) correction method. A highly significant list of DEGs (FC > 2.0 and p-adj <0.05) was obtained from the statistical analysis of T1 vs. T3, T1 vs. T4, T3 vs. T6 and T4 vs. T6 conditions (Figure 2(c)). The up- and down-regulation of these genes, as well as KEGG analysis, were performed (Figure 3). Subsequently, 1941 (T1 vs.T3), 2107 (T1 vs.T4), 1292 (T3 vs.T6) and 661 (T4 vs.T6) highly significant genes were identified, respectively. These lists were compared using the Venny web-application. A total of 128 genes were observed to be common across all comparisons, with 31 genes common among T1 vs. T3, T3 vs. T6 and T4 vs. T6 comparisons, 56 genes common among T1 vs. T3, T1 vs. T4 and T4 vs. T6 comparisons, 166 genes common among T1 vs. T3, T1 vs. T4 and T3 vs. T6 comparisons, and 69 genes common among T1 vs. T4, T3 vs. T6 and T4 vs. T6 comparisons (Figures 6(a)–1). Furthermore, statistical enrichment of DEGs in KEGG pathways resulted in a total of 10 significant pathways that were common among more than three statistical comparisons, respectively (Figures 6(a)–2).

### 3.3. Differentially expressed genes in mutant type (lcd/des1) Arabidopsis thaliana plants under different treatments

**Figure 4.**
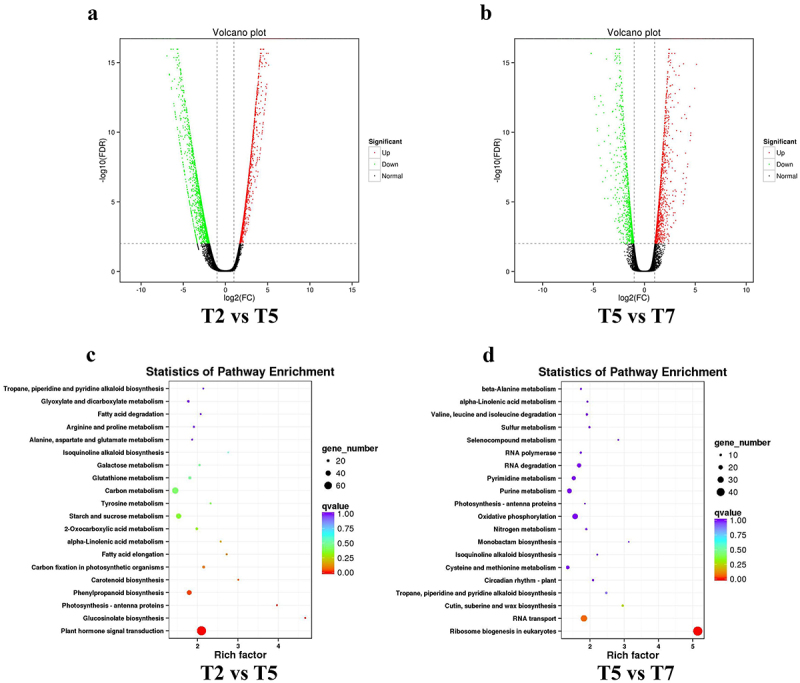
Significantly DEGs and their respective KEGG pathway analyses in *lcd/des1* mutants under both sole drought stress and a combined regulation involving drought stress and H₂S signaling.

To identify DEGs in mutant *A. thaliana* plants under drought stress (simulated by PEG) (Figure 2(c)), a total of 1963 and 556 highly significant genes were identified in the statistical analysis of T2 vs. T5 and T5 vs. T7, respectively. The up- and down-regulation, as well as KEGG analysis, are displayed in Figure 4. A comparison of the most significant genes revealed that 288 genes are shared between the T2 vs. T5 and T5 vs. T7 conditions (Figures 6b–1). Statistical enrichment analysis of DEGs in KEGG pathways identified a total of 20 significant KEGG pathways for each comparison, with 4 KEGG pathways common to both comparisons (Figures 6(b)–2).

### 3.4. Differentially expressed genes between WT and lcd/des1 Arabidopsis thaliana plants under different treatments

**Figure 5.**
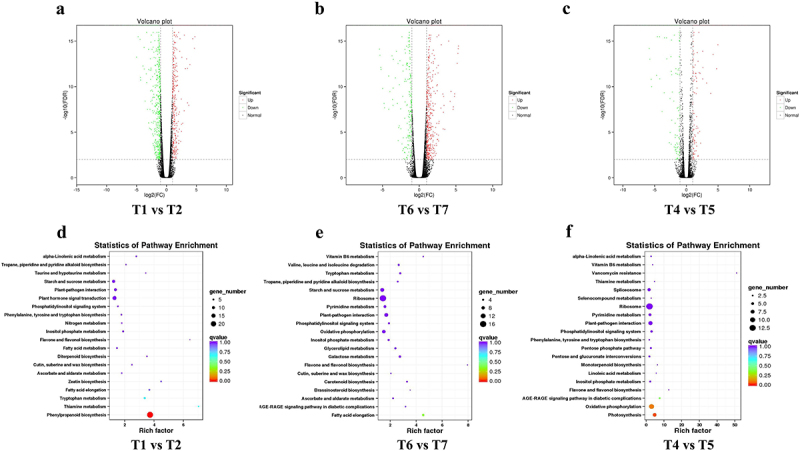
Significantly DEGs and their respective KEGG pathway analyses under various treatments for the wild-type (WT) and *lcd/des1* mutants.

**Figure 6.**
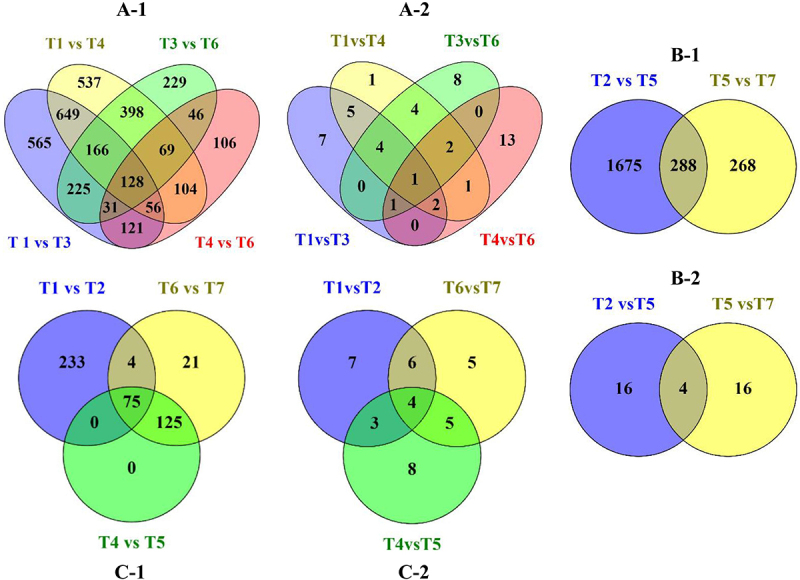
Comparison of DEGs (A-1, B-1, C-1) and KEGG pathways obtained from the statistical analysis (A-2, B-2, C-2).

The statistical analysis comparing conditions T1 vs. T2, T4 vs.T5 and T6 vs. T7 (Figure 2(c)) revealed 312, 200 and 225 DEGs respectively (Figures 6(c)–1). Comparison of these gene lists identified 75 common genes present across all datasets. Additionally, biological contextualization was performed using the KOBAS software to statistically enrich the DEGs in KEGG pathways. This enrichment resulted in 20 significant pathways for each set of DEGs derived from the above statistical analysis (Figure 5). Among the three datasets, a total of 4 common KEGG pathways were identified. Additionally, 5 pathways were common among T4 vs. T5 and T6 vs. T7 datasets, 6 pathways were common among T1 vs. T2 and T6 vs. T7 datasets, and 3 pathways were common between T1 vs. T2 and T4 vs. T5 datasets respectively (Figures 6(c)–2).

### 3.5. Differentially expressed genes under peg-induced drought stress and H_2_S treated samples

**Figure 7.**
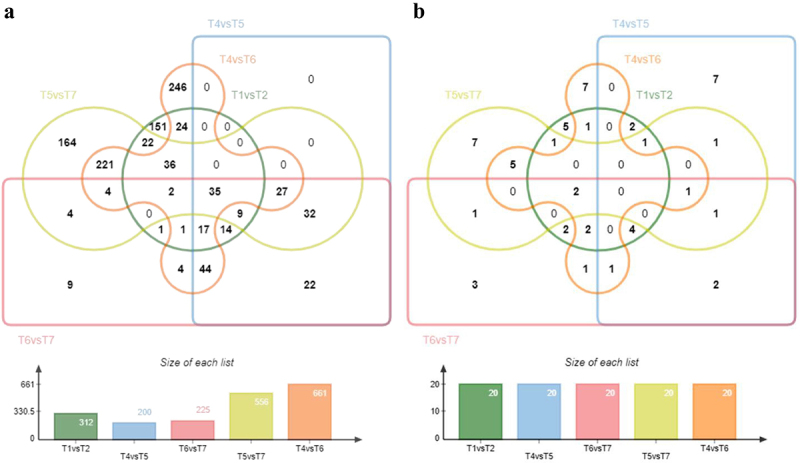
Five-way venn diagrams illustrating the comparison of DEGs and KEGG pathways obtained from the statistical analysis.

The DEGs resulting from pairwise comparisons among T1 vs. T2, T4 vs. T5, T6 vs. T7, T4 vs. T6, and T5 vs. T7 were individually analyzed (Figure 2(c)). The analysis identified a total of 35 genes shared across all the DEG sets (Figure 7(a)). Furthermore, a comparative analysis was conducted to evaluate the statistical enrichment of KEGG pathways associated with these DEGs. The comparisons between T1 vs. T2, T4 vs. T5, and T6 vs. T7 revealed four common KEGG pathways across all datasets, while eight common KEGG pathways were observed in the T4 vs. T6 and T5 vs. T7 comparisons, respectively (Figure 7(b)).

### 3.6. Validation of RNA-Seq results using qRT-pcr

**Figure 8.**
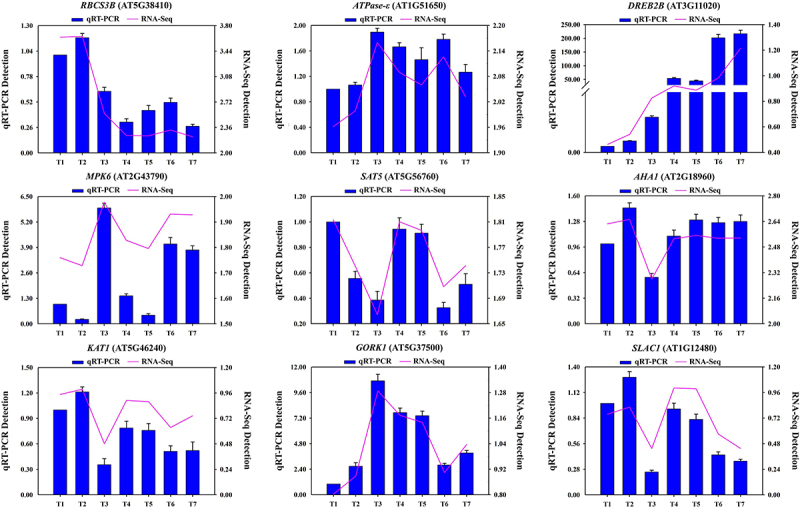
The transcriptional expression levels of 9 candidate genes were evaluated using qRT-PCR and RNA-Seq.The blue bars indicate the relative expression levels determined by qRT-PCR using the 2^−∆∆CT^ method, with associated standard error bar (*n* = 3). The red lines represent changes in transcript abundance from the RNA-Seq data, calculated using the Log10(FPKM +1) method. T1–7 on the X-axis denote different treatment groups: T1, WT; T2, *lcd/des1*; T3, WT+S; T4, WT+P; T5, *lcd/des1*+P; T6, WT+P+S; T7, *lcd/des1*+P+S; where P stands for PEG; S stands for H_2_S

In order to ascertain the reliability of the transcriptome data, nine DEGs were randomly selected for validation using quantitative real-time PCR (qRT-PCR). As illustrated in Figure 8, the nine DEGs chosen are involved in photosynthetic performance (*Ribulose 1,5-bisphosphatebisphosphate carboxylase/oxygenase small subunit 3B*, *RBCS3B*), ATP synthesis (*ATP synthase epsilon chain*, *ATPase-ε*), stress response (*Dehydration responsive element-binding factor 2B*, *DREB2B*; *Mitogen-activated protein kinases 6*, *MPK6*), cysteine synthesis (*Serine acetyltransferase 5*, *SAT5*), K^+^ channels (*K*^*+*^*channel of Arabidopsis thaliana*, *KAT1*; *Gated outwardly-rectifying K*^*+*^ *channel*, *GORK1*), anion channels (*Slow anion channel-associated 1*, *SLAC1*), and proton flow regulation (*Arabidopsis H*^*+*^*-ATPase 1*, *AHA1*). The expression patterns generally concurred with the transcriptome data (Figure 8; Table S4).

## 4. Discussion

Water is essential for the growth, development, and survival of plants. In nature environments, plants frequently encounter a variety of environmental stresses, with water scarcity being the significant factor affecting their growth and productivity.^46,47^ Throughout evolution, plants have developed intricate adaptive mechanisms to safeguard themselves against water scarcity. Over the last fifty years, researchers have recognized osmotic adjustments, antioxidant defenses, and stomatal movement as vital adaptive strategies that plants employ for survival.^46^ Osmotic stress in plants is commonly linked to other environmental stresses such as drought, soil salinity, and cold, all of which significantly affect plant productivity. Previous studies have shown that osmotic conditions trigger diverse signaling pathways that lead to changes in gene expression and plant metabolism. The *lcd* and *des1* genes of *A. thaliana* encode L-cysteine desulfhydrolase (LCD), which plays a critical role in cysteine degradation and H_2_S generation in cellular compartments.^2,48^ Mutations in the *lcd* and *des1* genes inhibit H_2_S generation in the cytosol, thereby strongly regulating plant metabolism.^2,48^ In this study, we conducted transcriptome sequencing of wild-type and double mutant (*lcd* and *des1*) *A. thaliana* plants exposed to drought stress induced by 40% PEG and 20 µM NaHS. Clean reads obtained after quality control analysis (> Q30) were aligned using the HiSeq2 tool, with an average of 95% uniquely mapped reads. Differential gene expression analysis was performed using the EBSeq statistical analysis method.

### 4.1. Differential expression of photosynthesis and antioxidant genes under drought stress

The comparison between wild-type and mutant-type plants treated with 40% PEG and 40% PEG +20 µM NaHS revealed 8 common genes related to KEGG pathways for photosynthesis, photosynthesis-antenna proteins and carbon fixation in photosynthetic organisms. Genes encoding photosystem I subunit D-1 (PSI-D), ATPase- F0 complex were highly expressed in T1, T4, T5 and T7 conditions, while the *photosystem I subunit G* gene was highly expressed in T1 and T7 conditions. Knockout studies of the *psad1*-1 and *psad2*-1 genes in *A. thaliana* showed defects in the accumulation of thylakoid protein complexes, highlighting the crucial role of PSI-D in photosynthesis.^49^ Additionally, the genes encoding for ferredoxin1, OrfB, ATP synthase CF0B subunit were significantly up-regulated in samples T4, T5, and T7. In contrast, sample T2 exhibited downregulation of genes related to the *light-harvesting complex photosystem II*. Similarly, the genes encoding for ribose 5-phosphate isomerase-A and aldolase were highly expressed in T5, T6, and T7 samples respectively. PEG-induced drought stress mainly causes dehydration, leading to stomatal closure to prevent water loss, as the primary drought avoidance mechanism by plants.^50^ Prior research has indicated that stomatal closure disrupts the supply of carbon dioxide to parenchymal cells, significantly affecting photosynthetic efficiency and carbon assimilation.^51^ Furthermore, existing studies demonstrate that increased oxidative stress in plants leads to a decline in chlorophyll levels due to pigment photo-oxidation and oxidative stress-induced degradation.^52^ Additionally, drought stress in plants causes to degradation of photosynthetic pigments, which has a negative impact on the entire photosynthetic apparatus.^53^ The results indicate that H_2_S signaling plays a crucial role in modulating photosynthesis reaction systems under drought stress conditions. The upregulation of key photosynthesis-related genes in both wild type and mutant plants with a functional H_2_S signaling pathway provides further support for this conclusions.

### 4.2. Differentially expressed pathways in wild and mutant plants subjected to PEG induced drought stress and H_2_S treatment

This study compared the statistically significant and enriched pathways between experimental conditions T1 vs. T2, T4 vs. T5, and T6 vs. T7. Notably, the KEGG pathways pertaining to inositol phosphate metabolism, flavone and flavanol biosynthesis, plant-pathogen interaction, and phosphatidylinositol signaling system consistently emerged as significant across all three comparative groups. Furthermore, we compared the list of DEGs from T1 vs. T2, T4 vs. T5, and T6 vs. T7 involved in these KEGG pathway. Threonine aldolase 1, photosystem I subunit G, uncharacterized protein family (UPF0497), and disease resistance-responsive protein were common among T1 vs. T2 and T6 vs. T7. Glycosyltransferase 1 was highly expressed in T4 vs. T5 and T6 vs. T7 datasets, respectively. All of the mentioned genes were highly expressed in the T2, T5, and T7 samples. Inositol signaling plays a crucial role in plant growth and environmental adaptation.^54^ Previous studies have reported that under environmental abiotic stress, plant cell membranes respond to stimuli by passing cellular messages through phosphoinositides on the membrane, generating secondary messengers such as diacylglycerol and inositol triphosphate (IP_3_).^55^ In wheat, overexpression of TaVIH2-3B, a high-energy inositol derivative, enhances drought stress tolerance.^56^ Similarly, inositol-1-phosphate synthase genes within the Rosaceae family exhibit significant roles in plant growth, development, and stress adaptation.^57^ A prior study extensively investigated drought stress using time-series experiments in conjunction with untargeted metabolomic analysis, resulting in the successful identification of ten flavonoids compounds. These findings emphasized the sensitivity and responsiveness of flavonoids to drought stress.^58^ Additionally, another study disclosed that drought-tolerant citrus varieties, including sour oranges, possess the ability to synthesize flavonoids with augmented antioxidant properties via distinct biosynthetic routes. These flavonoids mitigated the adverse impacts arising from reactive oxygen species during drought stress, thereby protecting plants.^59^ Studies have reported that abiotic and biotic stresses influence the expression of genes related to plant-pathogen interaction pathways both positively and negatively in plants, thus increasing or decreasing the severity of the disease conditions.^60^ Previous studies have shown that plants exposed to drought/salinity stress exhibit a strong association of the abiotic and biotic stress, which facilitates plant disease progression.^58^ Additionally, plants under abiotic stress are more prone to being affected by plant pathogens. However, further studies are needed to reveal the interactions between abiotic and biotic stress with plant-pathogen interaction pathways. Genes involved in isoquinoline alkaloid biosynthesis exhibited significant up-regulation in T2 vs. T5 and T5 vs. T7 conditions, especially in mutant plant samples, followed by wild type plants. A transcriptomic study of the desert plant *Populus euphratica* conducted by Qiu et al. (2011) has revealed significant expression of various plant secondary metabolite pathways, including isoquinoline alkaloid biosynthesis, as a drought-responsive mechanism.^60^ Moreover, the results have shown that genes involved in the phenylpropanoid pathway were differentially expressed in samples treated with PEG and NaHS. The phenylpropanoid pathway was found to be common among in the KEGG pathways of T1 vs. T2, T2 vs. T5 and T4 vs. T6 conditions. Phenylpropanoid metabolism, a pivotal metabolic pathway in plants, coordinates growth and development, mediates plant-environment interactions, and modulates metabolic fluxes among various pathways.^61^

The α-linolenic acid, a precursor of the jasmonate pathway, plays a vital role in plant defense mechanisms. Mata-Mata-Pérez et al., conducted a transcriptome study on *A. thaliana* plants exposed to reactive oxygen species supplemented with linolenic acid, suggesting that linolenic acid may trigger a defense response involving key components like protein stabilizers, particularly in responses to abiotic stresses such as drought/salinity.^62^ The assessment of gene expression revealed significant levels in the statistical comparisons of T1 vs. T2, T2 vs. T5, T4 vs. T5, and T5 vs. T7 for genes associated with the α-linolenic acid pathway. Additionally, genes involved in the ribosome pathway (T4 vs. T5, T6 vs. T7, and T4 vs. T6) and the oxidative phosphorylation KEGG pathway (T5 vs. T7, T4 vs. T5, and T6 vs. T7) exhibited considerable expression, underscoring their significance in plants under drought/salinity stress conditions. Additionally, Alqurashi et al., conducted a proteomic study on *A. thaliana* plants subjected to severe drought stress, reporting changes in genes related to GO biological functions such as ‘response to water deprivation’ and ‘response to osmotic stress’, as well as moderate changes in genes related to ‘vesicle-mediated transport’, ‘ribosome biogenesis’ and ‘oxidative phosphorylation’.^63^ The overexpression of genes involved in ribosomal biogenesis and components associated with oxidative phosphorylation during early plant growth under drought stress suggests the molecular machinery involved in *de novo* protein synthesis is an essential defense mechanism.^63^ The AGEs (advanced glycation end-products)/RAGE (AGE containing peptides and receptors) were found to play important roles in cellular signaling mechanisms by triggering defense pathways. The binding of AGE peptides leads to pro-inflammatory responses, production of ROS, NADPH-oxidase activation and MAPK (mitogen-activated protein kinases) signaling pathways.^64,65^ Similarly, vitamin B6 metabolism pathway plays a crucial role in initiating plant defense response mechanisms against oxidative and abiotic stress. Previous studies have reported that *de novo* vitamin B6 biosynthetic genes *PDX1* and *PDX2* were highly expressed in *A. thaliana* plants under different abiotic stresses.^66^ The findings from this research indicate that the AGE/RAGE signaling pathway and vitamin B6 metabolic signaling were significantly upregulated and consistently noted across the conditions T4 vs. T5 and T6 vs. T7, particularly in mutant plants exposed to 40% PEG and 20 µM NaHS.

## 5. Conclusion

**Figure 9.**
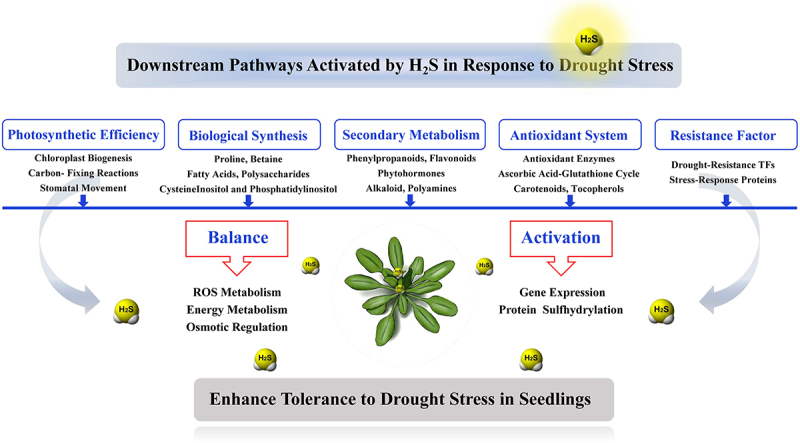
A model depicting the regulatory pathways mediated by H_2_S in enhancing drought tolerance in *Arabidopsis* seedlings.

This investigation performed transcriptome analysis on both wild type and mutant (*lcd/des1) A. thaliana* plants under PEG-induced drought stress to elucidate the role of H_2_S signaling in drought-responsive mechanisms. The findings indicated that wild type plants exhibit enhanced drought tolerance attributed to active H_2_S signaling, whereas mutants lacking functional *lcd/des1* genes, crucial for H_2_S synthesis, exhibited heightened sensitivity to PEG-induced drought stress compared to mutants treated with 20 µM NaHS. Differential gene expression analysis identified key genes implicated in photosynthesis, biosynthesis, regulation of various plant secondary metabolites, defense mechanisms, immunity, and stress responses, all of which were significantly overexpressed. The statistical enrichment of KEGG pathways has revealed that a) the phenylpropanoid pathway, b) flavones and flavanols biosynthesis, c) isoquinoline alkaloid synthesis, d) α-linolenic acid biosynthesis pathway, e) ribosome, f) oxidative phosphorylation, g) plant-pathogen interactions, and h) inositol phosphates metabolism, etc., were commonly enriched across all datasets. Based on the above analysis, a schematic diagram of the downstream signaling pathway triggered by H_2_S during plant response to drought stress has been constructed (Figure 9). This provides a reference for comprehensively understanding the stress resistance mechanism of the signaling molecule H_2_S.

## Supplementary Material

Supplementary Materials.docx
