# Supplementary material for: Elucidating the downstream pathways triggered by H2S signaling in Arabidopsis thaliana under drought stress via transcriptome analysis
Source: Plant Signal Behav. 2024 Oct 4;19(1):2411911. doi: 10.1080/15592324.2024.2411911 (PMC11457601; doi:10.1080/15592324.2024.2411911)
Supplement: Supplementary Materials.docx [file KPSB_A_2411911_SM9615.docx]

**Elucidating the downstream pathways triggered by H_2_S signaling in *Arabidopsis thaliana* under drought stress via transcriptome analysis**

**Table S1.** Illustrates the results obtained from the transcriptome sequencing of *Arabidopsis thaliana* samples

| **Sample Name** | **Total Read** | **Clean Read** | **Clean bases** | **GC Content** | **%≥Q30** |
| --- | --- | --- | --- | --- | --- |
| Arab_P091-01-T01 | 26616458 | 26162882 | 7,772,885,816 | 45.52% | 94.79% |
| Arab_P091-01-T02 | 30401577 | 29952839 | 8,899,030,074 | 45.36% | 95.35% |
| Arab_P091-01-T03 | 32047043 | 31565491 | 9,375,178,760 | 45.18% | 94.92% |
| Arab_P091-01-T04 | 32262948 | 31873101 | 9,488,471,726 | 45.14% | 95.08% |
| Arab_P091-01-T05 | 21015538 | 20764521 | 6,177,139,022 | 45.36% | 94.77% |
| Arab_P091-01-T06 | 24038578 | 23692517 | 7,045,749,936 | 45.28% | 95.13% |
| Arab_P091-01-T07 | 20010201 | 19821366 | 5,919,719,764 | 45.14% | 94.64% |

**Table S2.** Alignment results obtained for the *Arabidopsis thaliana* transcriptome sequenced samples

| **Sample Name** | **Total Reads** | **Mapped** | **Unique Mapped** | **Multiple Mapped** | **Mapped to '+'** | **Mapped to '-'** |
| --- | --- | --- | --- | --- | --- | --- |
| Arab_P091-01-T01 | 52,325,764 | 51,113,75 (**97.68%**) | 49,852,196 **(95.27%)** | 1,261,509 **(2.41%)** | 25,469,171 **(48.67%)** | 25,505,244 **(48.74%)** |
| Arab_P091-01-T02 | 59,905,678 | 58,693,561 (**97.98%)** | 57,133,693 **(95.37%)** | 1,559,868 **(2.6%)** | 29,235,022 **(48.8%)** | 29,272,570 **(48.86%)** |
| Arab_P091-01-T03 | 63,130,982 | 61,805,672 **(97.9%)** | 60,332,544 **(95.57%)** | 1,473,128 **(2.33%)** | 30,770,041 **(48.74%)** | 30,835,449 **(48.84%)** |
| Arab_P091-01-T04 | 63,746,22 | 62,454,077 **(97.97%)** | 60,974,478 **(95.65%)** | 1,479,599 **(2.32%)** | 31,128,840 **(48.83%)** | 31,179,473 **(48.91%)** |
| Arab_P091-01-T05 | 41,529,042 | 40,591,393 **(97.74%)** | 39,634,379 **(95.44%)** | 957014 **(2.3%)** | 20,215,334 **(48.68%)** | 20,251,696 **(48.77%)** |
| Arab_P091-01-T06 | 47,385,034 | 46,436,580 **(98%)** | 44,986,323 **(94.94%)** | 1,450,257 **(3.06%)** | 23,126,487 **(48.81%)** | 23,168,725 **(48.89%)** |
| Arab_P091-01-T07 | 39,642,732 | 38,831,162 **(97.95%)** | 37,668,07 **(95.02%)** | 1,163,155 **(2.93%)** | 19,336,890 **(48.78%)** | 19,368,659 **(48.86%)** |

**Table S3.** Annotation of the transcriptome results obtained from the different annotation databases were listed below:

| **Anno Database** | **Annotated Number** | **300 ≤ length< 1000** | **Length≥ 1000** |
| --- | --- | --- | --- |
| COG | 9002 | 1290 | 7665 |
| GO | 26016 | 7130 | 18312 |
| KEGG | 8319 | 1775 | 6434 |
| KOG | 13630 | 2940 | 10521 |
| Pfam | 21008 | 4625 | 16217 |
| Swiss-Prot | 20254 | 4825 | 15103 |
| eggNOG | 23936 | 5830 | 17833 |
| NR | 27627 | 7959 | 18824 |
| Total | 27659 | 7984 | 18825 |

**Table S4****.** Primers used for quantitative real-time PCR (qRT-PCR).

| **Target** | **Aliases** | **Primer name** | **Primer sequence (5’ To 3’)** |
| --- | --- | --- | --- |
| *RBCS3B* | AT5G38410 | Forward primer | CTGCTCATTTCATTTCCTATTG |
|  |  | Reverse primer | TCATAAATCAGACATTTGACAATC |
| *ATPase-ε* | AT1G51650 | Forward primer | ATGCGGCGGTTCCGTTCT |
|  |  | Reverse primer | AGGTTTCTGGGGCTTTCC |
| *DREB2B* | AT3G11020 | Forward primer | GAACAAACCGGAACCGAGC |
|  |  | Reverse primer | GCGTTTCGGTTTCTCTCCTTC |
| *MPK6* | AT2G43790 | Forward primer | GCGGCTCCATCGCCTCAGAT |
|  |  | Reverse primer | ACGATGCCATAAGCACCCTTGC |
| *SAT5* | AT5G56760 | Forward primer | AAGATTGGTGCAGGTGCTA |
|  |  | Reverse primer | TCCGAGATGAATGAAGTATG |
| *AHA1* | AT2G18960 | Forward primer | GGTCTTAGGTCGTTGGCAGTA |
|  |  | Reverse primer | TGTCGTGTCTTGGAGGGTC |
| *KAT1* | AT5G46420 | Forward primer | TGAAACTTAGAGGGCAACA |
|  |  | Reverse primer | GAATCGCATCCATTAGAGC |
| *GORK1* | AT5G37500 | Forward primer | GTCGTCTATCTACCCGTCAA |
|  |  | Reverse primer | ACAACTCCGTGCTTTCTACT |
| *SLAC1* | AT1G12480 | Forward primer | ACCGAGGGAAACAAAGACC |
|  |  | Reverse primer | CTGATTCAAACCCGCCAAA |
| *Actin* | AT3G46520 | Forward primer  Reverse primer | GGGCACTCAAGTATCTTGTTAGC  TGCTGCCCAACATCAGGTT |
